# Chronic breathlessness: re-thinking the symptom

**DOI:** 10.1183/13993003.02331-2017

**Published:** 2018-01-25

**Authors:** Jane Macnaughton, Rebecca Oxley, Arthur Rose, Andrew Russell, James W. Dodd, Havi Carel

**Affiliations:** 1 Centre for Medical Humanities, Durham University, Durham, UK; 2 Dept of Anthropology, Durham University, Durham, UK; 3 Life of Breath Project, Durham, UK; 4 Dept of English Studies, Durham University, Durham, UK; 5 Dept of Anthropology, Durham University, Durham, UK; 6 Academic Respiratory Unit, Southmead Hospital, Bristol, UK; 7 Respiratory Medicine, University of Bristol, Bristol, UK; 8 Life of Breath Project, Bristol, UK; 9 Dept of Philosophy, University of Bristol, Bristol, UK

## Abstract

We wholly applaud the move by Johnson *et al.* [1] to improve awareness of breathlessness and to raise its profile as a subject for focussed clinical research. We consider their research and the ensuing proposal to recognise breathlessness *via* a new medical term, “chronic breathlessness syndrome”, as important and justified. We share their goal, which is to direct attention to this neglected, undertreated and under-researched symptom.

## Abstract 2

**People with breathlessness should be involved in renaming their condition as their experience can be influenced by clinical language** http://ow.ly/ZKNj30hzUUM

To the Editor:

We wholly applaud the move by Johnson *et al.* [1] to improve awareness of breathlessness and to raise its profile as a subject for focussed clinical research. We consider their research and the ensuing proposal to recognise breathlessness *via* a new medical term, “chronic breathlessness syndrome”, as important and justified. We share their goal, which is to direct attention to this neglected, undertreated and under-researched symptom.

There are two important caveats to be made in response to this article, however. First, there is a need to involve those who live with chronic breathlessness and are thus “experts by experience” in discussions about the framework proposed here, rather than bringing them into the conversation once consensus has been achieved. Second, further medicalisation of breathlessness *via* the term “syndrome” may not be the best way forward. Research into patients' experience of breathlessness shows that the ways in which breathlessness is spoken about (medicalised and otherwise) not only reflect their experiences but also helps to shape how breathlessness is lived [2–6].

O.K. Faull and colleagues, in their response to Johnson *et al.* [1], comment on the individuality of responses to breathlessness that rely on prior experiences and bodily awareness (interoception). Context and culture play an important role in shaping the understanding and perception of breathlessness [2–4, 6]. For example, among African American communities across the USA, the last words of Eric Garner, “I can't breathe”, as he suffocated in a tussle with police officers, have become a slogan for the Black Lives Matter movement and a metaphor for the lives of those living under other kinds of oppression [2]. Başoğlu [7] suggests that asphyxiation is the most traumatic form of torture and that persistent breathlessness because of an underlying medical condition may be even worse due to the duration of the suffering involved.

Sufferers of respiratory illness vary in relation to the intensity, affect, ideation and meaning they attribute to their breathlessness [3–6]. It affects every aspect of the life of a breathless person in ways that description of it as a medical symptom cannot capture in full [8]. There is a need to legitimise a range of attitudes towards breathlessness in order for them to inform the clinical encounter. Collecting such experiences under the umbrella term “syndrome” may not be sufficient to enable full expression of the variability and multiple meanings of the experience of breathlessness, and may carry unexpected cognitive and affective “baggage” that detracts from its utility as a proxy for experience.

In view of the highly contextualised experience of breathlessness, it is critical to think about whose views are part of the debate. Discussions with experts by experience and first-person reports of experiences of breathlessness [3, 5] have revealed how powerful language and context are in determining how people with breathlessness think about and experience their problem [4, 6], and how this influences what they might do. Words such as “pulmonary” and “rehabilitation”, for example, may negatively impact upon the uptake of one of the most effective interventions for breathlessness [6].

There is a further stage necessary in the research process of Johnson *et al.* [1] in order to validate the claim made in the paper that “a recognised syndrome would […] give permission for patients to discuss their ongoing breathlessness with their clinicians”. As Johnson *et al.* [1] suggest, patients and their families need to be involved in the discussion, but they should be able to critique the framework suggested by the paper, rather than be presented with it as a *fait accompli*. Otherwise there is a danger that the words “chronic” and “syndrome” will drive people with breathlessness further underground, in part because they have not been involved in the process of describing their own condition [9]. We encourage Johnson *et al.* [1] to take this research on to its next logical stage, that of developing a truly consensual terminology that considers the critical role language, metaphor and meaning play in both living with and treating breathlessness. This could be done using the Delphi technique within a more participatory paradigm [10]. Such an approach offers the chance of empowering patients and caregivers in ways that would result in real changes to both their experience and treatment.

## Disclosures

10.1183/13993003.02331-2017.Supp1 H. Carel ERJ-02331-2017_Carel
J.W. Dodd ERJ-02331-2017_Dodd
J. Macnaughton ERJ-02331-2017_MacNaughton
R. Oxley ERJ-02331-2017_Oxley
A. Rose ERJ-02331-2017_Rose
A. Russell ERJ-02331-2017_Russell

## References

[1] Johnson MJ, Yorke J, Hansen-Flaschen J, et al. Towards an expert consensus to delineate a clinical syndrome of chronic breathlessness. Eur Respir J 2017; 49: 1602277. 28546269 10.1183/13993003.02277-2016

[2] Rose A Tim Winton's pneumatic materialism. Interventions 2017; in press. 10.1080/1369801X.2020.1715819 PMC7484908 32982570

[3] Carel H Phenomenology of Illness. Oxford, Oxford University Press, 2016.

[4] Oxley R, Macnaughton J Inspiring change: humanities and social science insights into the experience and management of breathlessness. Curr Opin Support Palliat Care 2016; 10: 256–261. 27490147 10.1097/SPC.0000000000000221 PMC4974063

[5] Carel H Illness: the cry of the flesh. 2nd Edn Durham, Acumen, 2013.

[6] Oxley R Lost in Translation? Exploring the Language of Breathlessness. Life of Breath blog https://lifeofbreath.org/2017/09/lost-in-translation-exploring-the-language-of-breathlessness/ Date last accessed: November 6, 2017. Date last updated: September 1, 2017.

[7] Başoğlu M Effective management of breathlessness: a review of potential human rights issues. Eur Respir J 2017; 49: 1602099. 28546267 10.1183/13993003.02099-2016

[8] Hayen A, Herigstad M, Pattinson KTS Understanding dyspnea as a complex individual experience. Maturitas 2013; 76: 45–50. 23849705 10.1016/j.maturitas.2013.06.005

[9] Kidd IJ, Carel H Epistemic injustice and illness. J Appl Philos 2016; 34: 172–190. 28303075 10.1111/japp.12172 PMC5324700

[10] Kezar A, Maxey D The Delphi technique: an untapped approach of participatory research. Int J Soc Res Methodol 2016; 19: 143–160.

